# Supplementary material for: Smith–Magenis syndrome protein RAI1 regulates body weight homeostasis through hypothalamic BDNF-producing neurons and neurotrophin downstream signalling
Source: eLife. 2023 Nov 13;12:RP90333. doi: 10.7554/eLife.90333 (PMC10642964; doi:10.7554/eLife.90333)

Uncropped western blots for staining against antibodies for P-AKT & AKT. The membrane was stripped to stain for AKT after staining for p-AKT. Before staining, the membrane was cut at band 72KDa and below 53 KDa. The yellow box represents the cropped region for images shown in this manuscript. For staining against H3, membrane was first cut below band ~23kD.

Figure 5

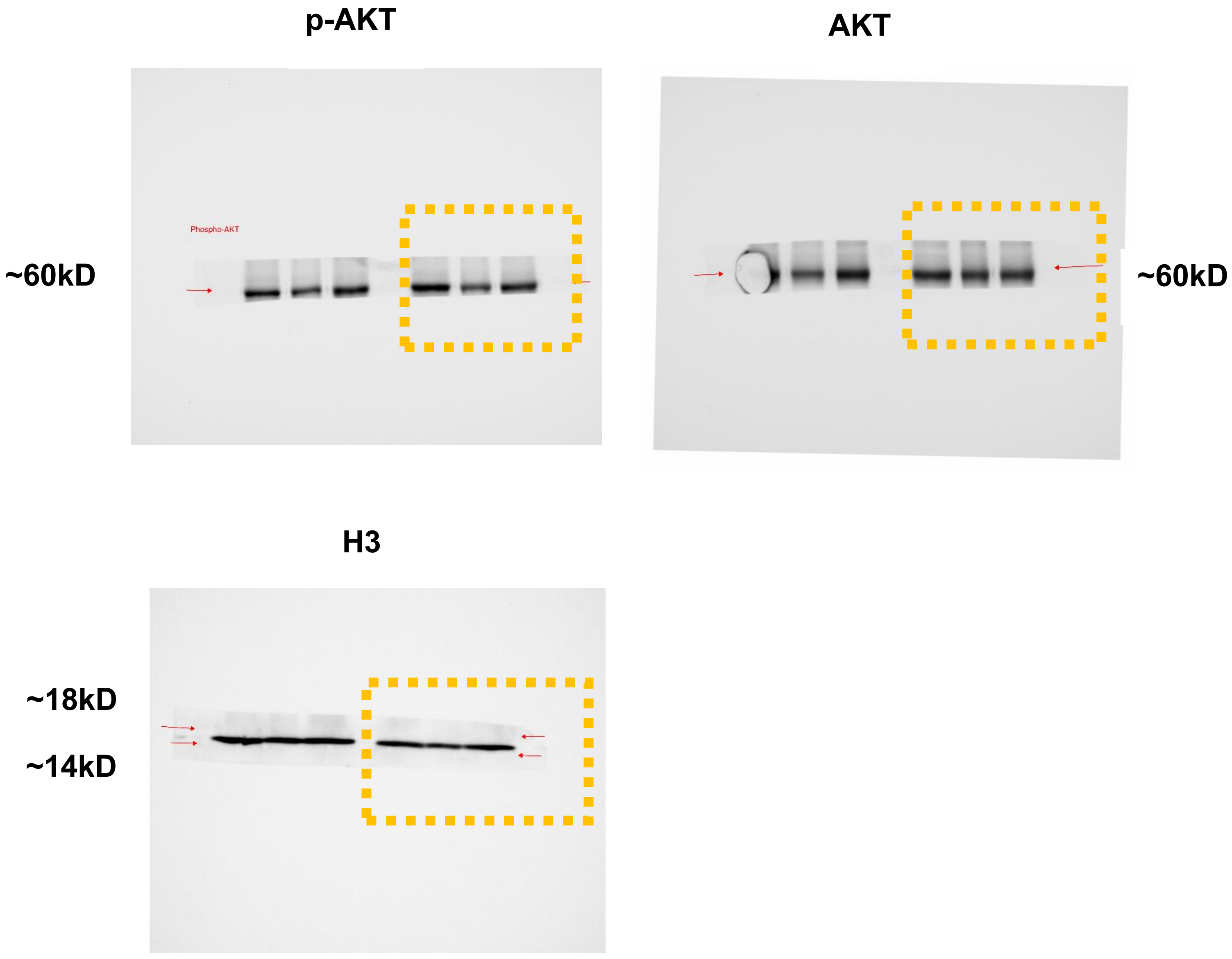

Supplement: Figure 5—source data 1. [file elife-90333-fig5-data1.zip › Figure 5-source data 1/Figure 5-source data 1.pdf]
