# Supplementary figures and images for: Smith–Magenis syndrome protein RAI1 regulates body weight homeostasis through hypothalamic BDNF-producing neurons and neurotrophin downstream signalling
Source: eLife. 2023 Nov 13;12:RP90333. doi: 10.7554/eLife.90333 (PMC10642964; doi:10.7554/eLife.90333)

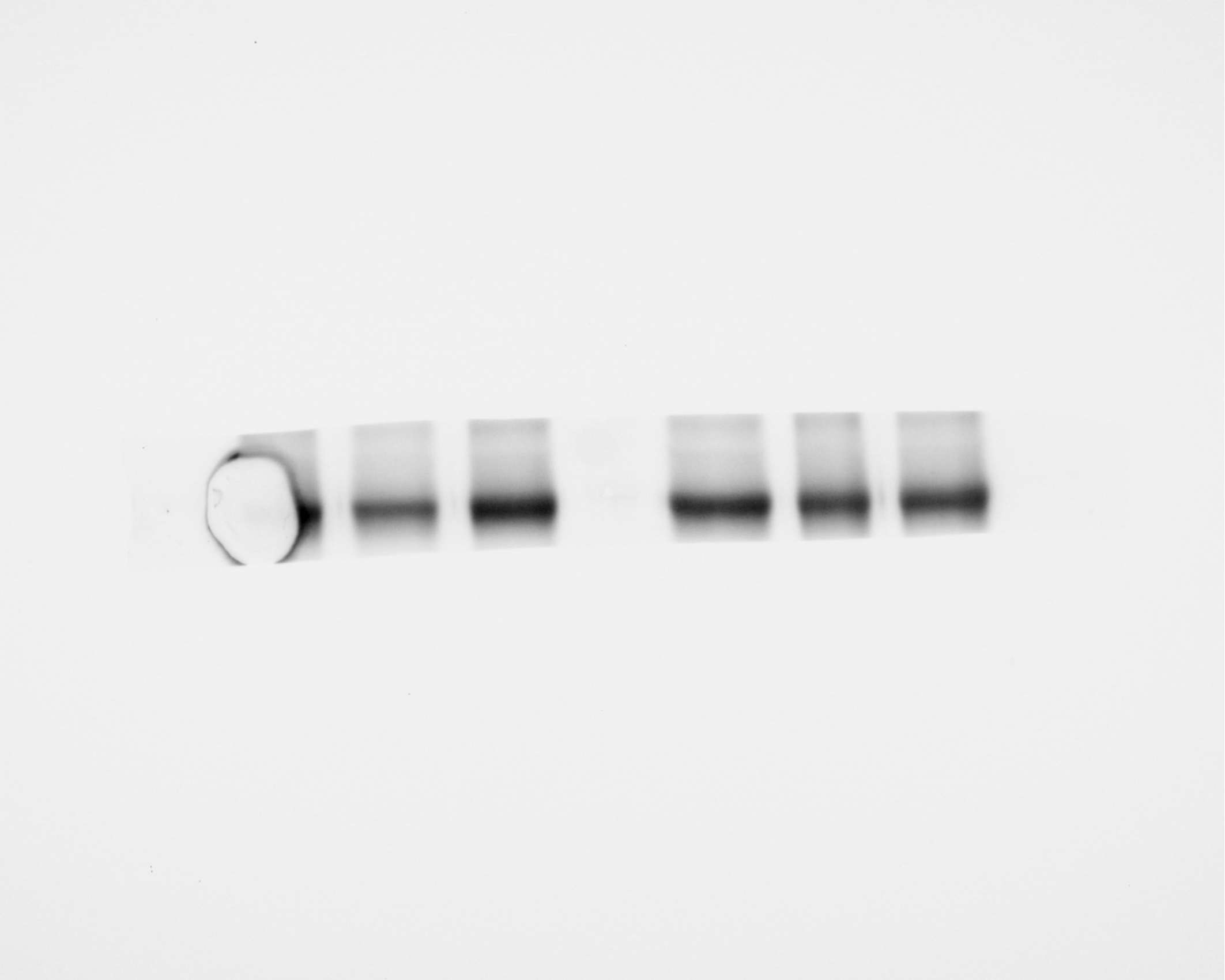

Supplement: Figure 5—source data 1. [file elife-90333-fig5-data1.zip › Figure 5-source data 1/Figure 5A AKT.jpg]

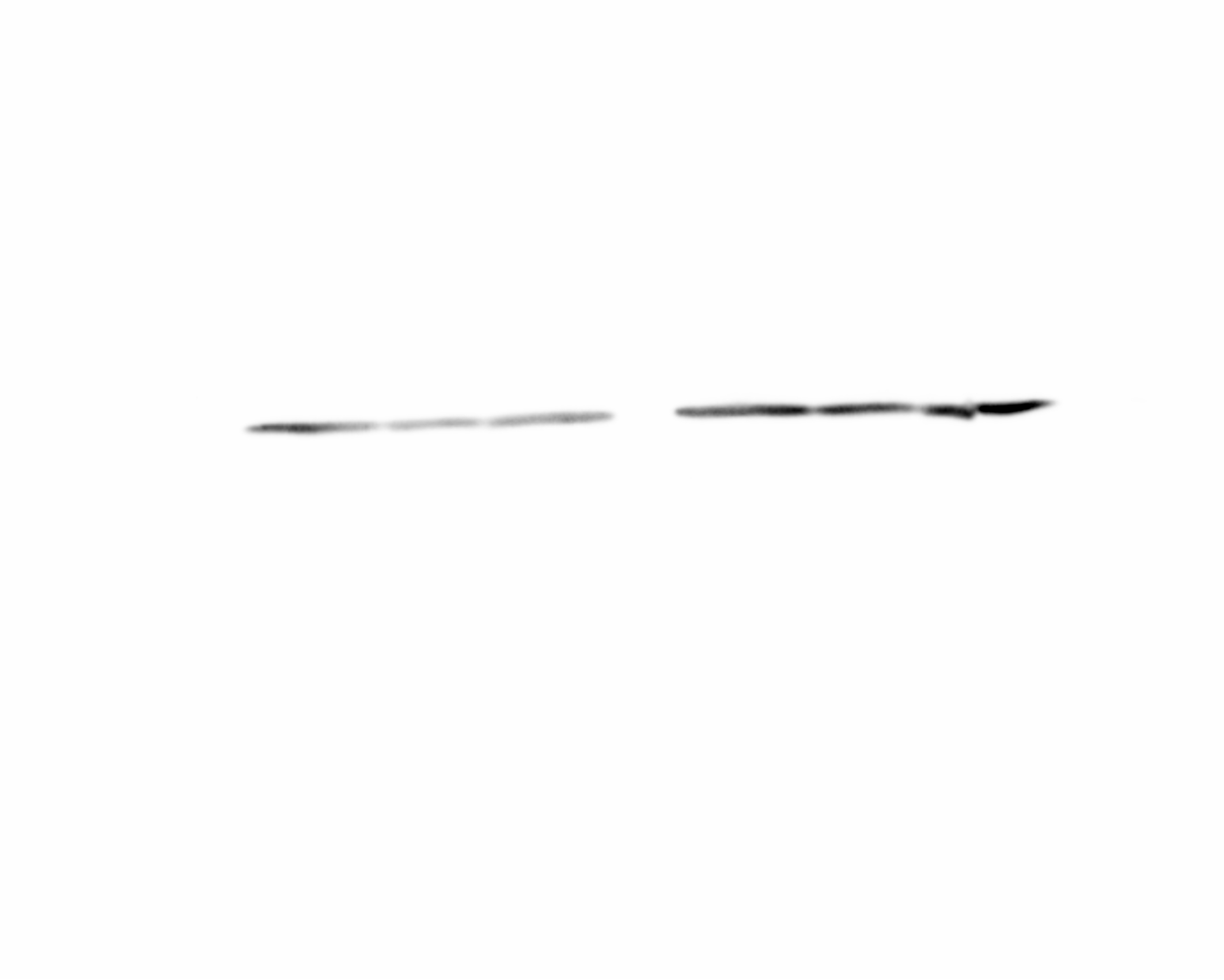

Supplement: Figure 5—source data 1. [file elife-90333-fig5-data1.zip › Figure 5-source data 1/Figure 5A H3.jpg]

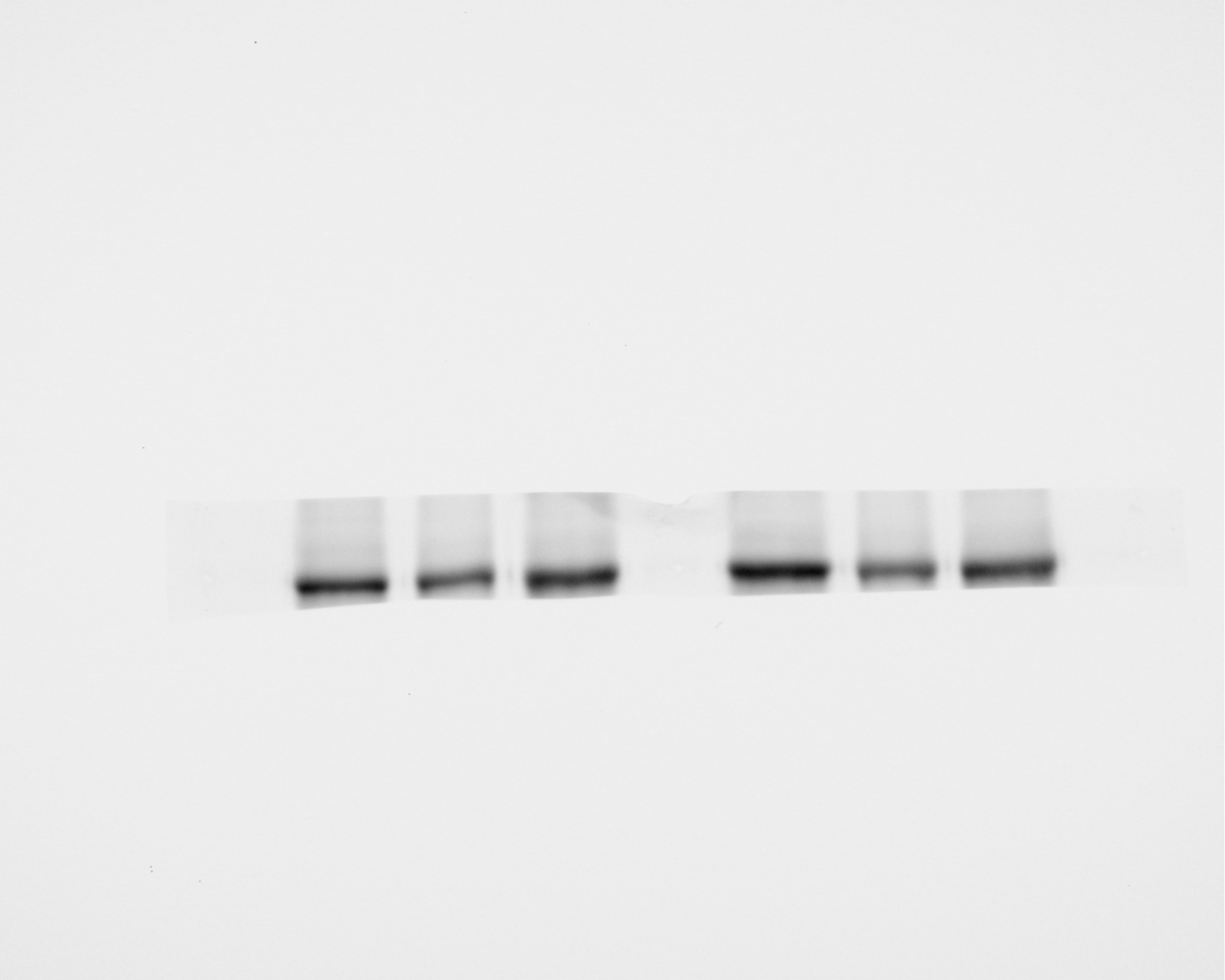

Supplement: Figure 5—source data 1. [file elife-90333-fig5-data1.zip › Figure 5-source data 1/Figure 5A p-AKT.jpg]

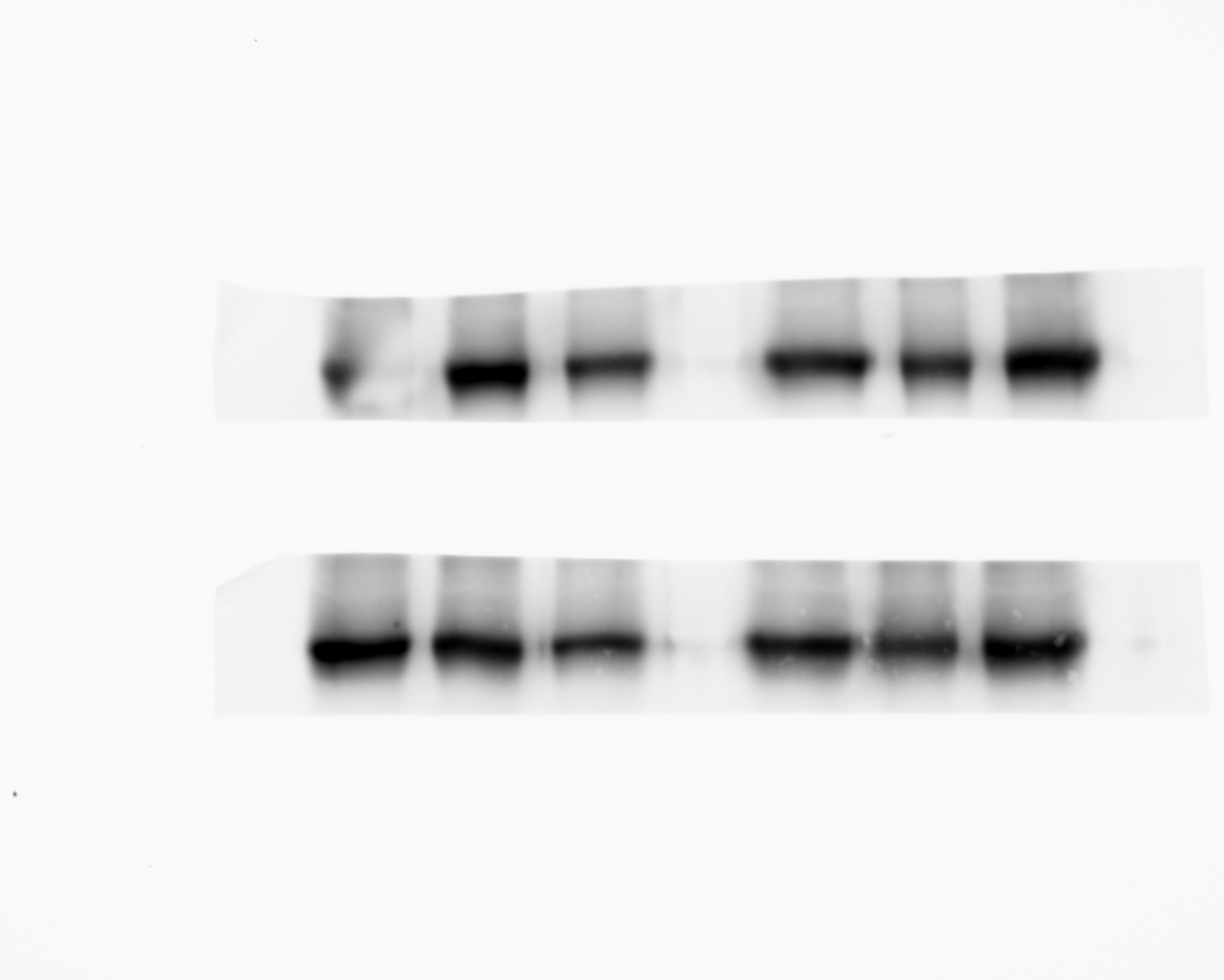

Supplement: Figure 5—figure supplement 1—source data 1. [file elife-90333-fig5-figsupp1-data1.zip › Figure 5-figure supplement 1 source data 1/Set 1-2/Set 1 and 2 AKT.jpg]

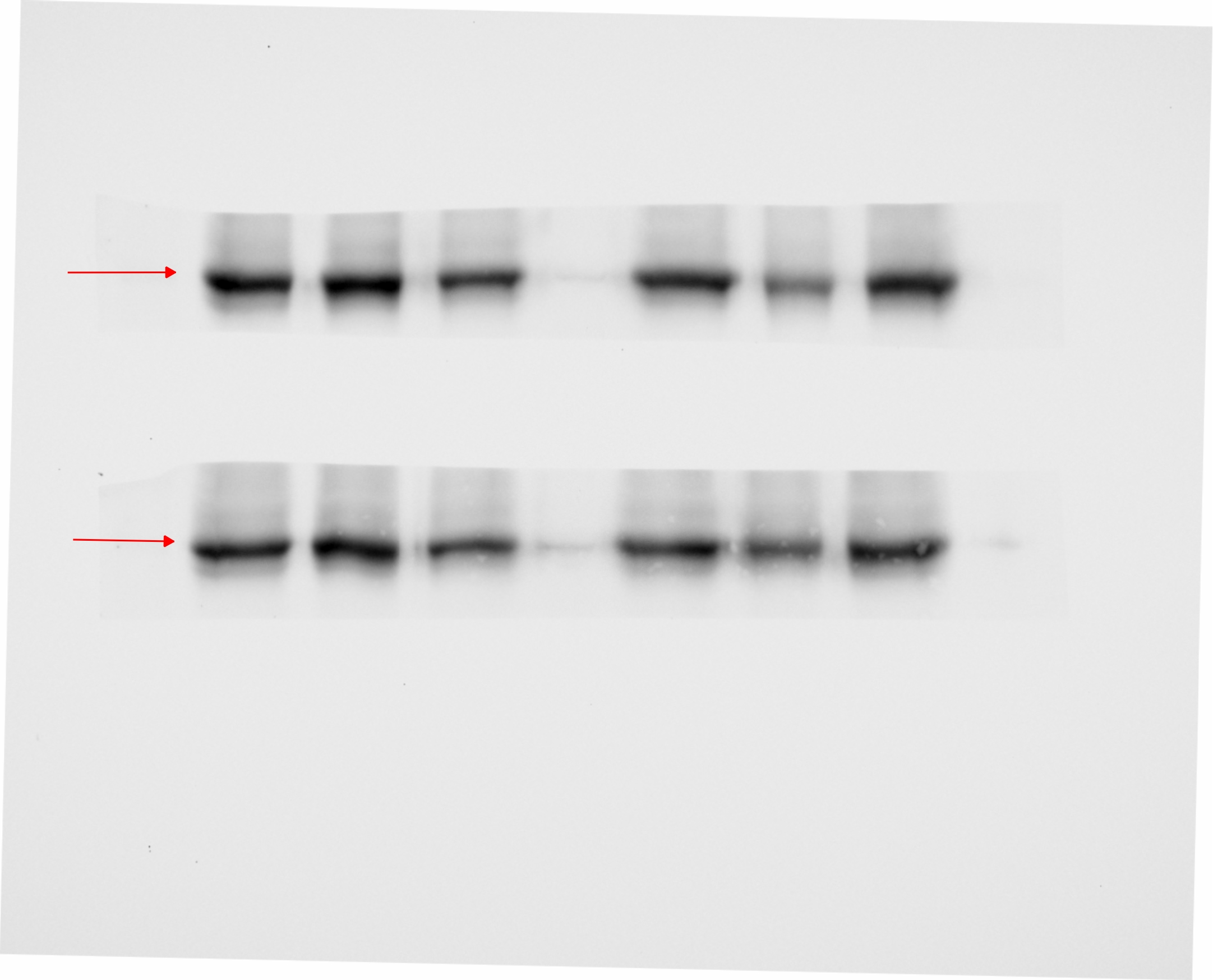

Supplement: Figure 5—figure supplement 1—source data 1. [file elife-90333-fig5-figsupp1-data1.zip › Figure 5-figure supplement 1 source data 1/Set 1-2/Set 1 and 2 p-AKT.jpg]

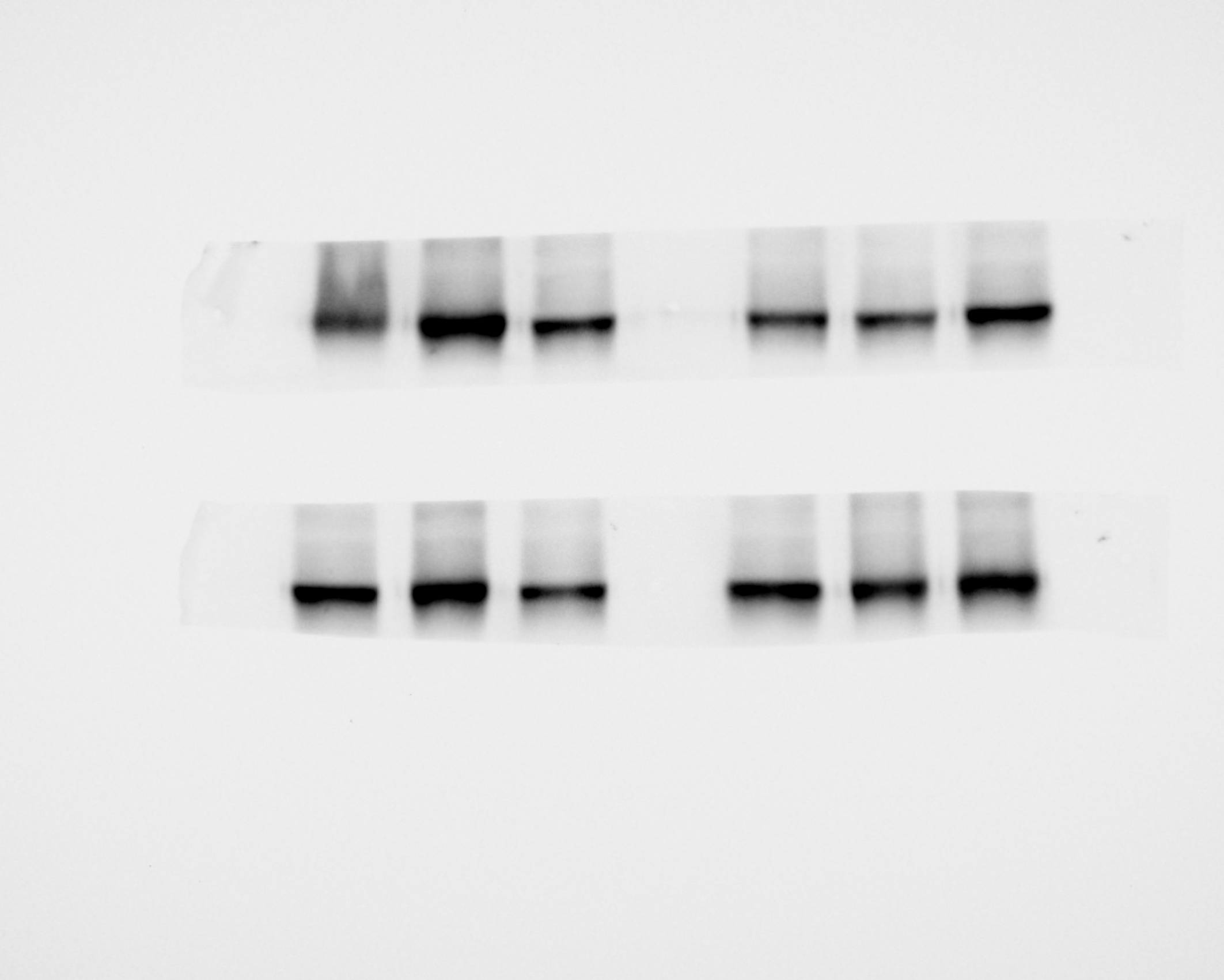

Supplement: Figure 5—figure supplement 1—source data 1. [file elife-90333-fig5-figsupp1-data1.zip › Figure 5-figure supplement 1 source data 1/Set 4/Set 4 AKT.jpg]

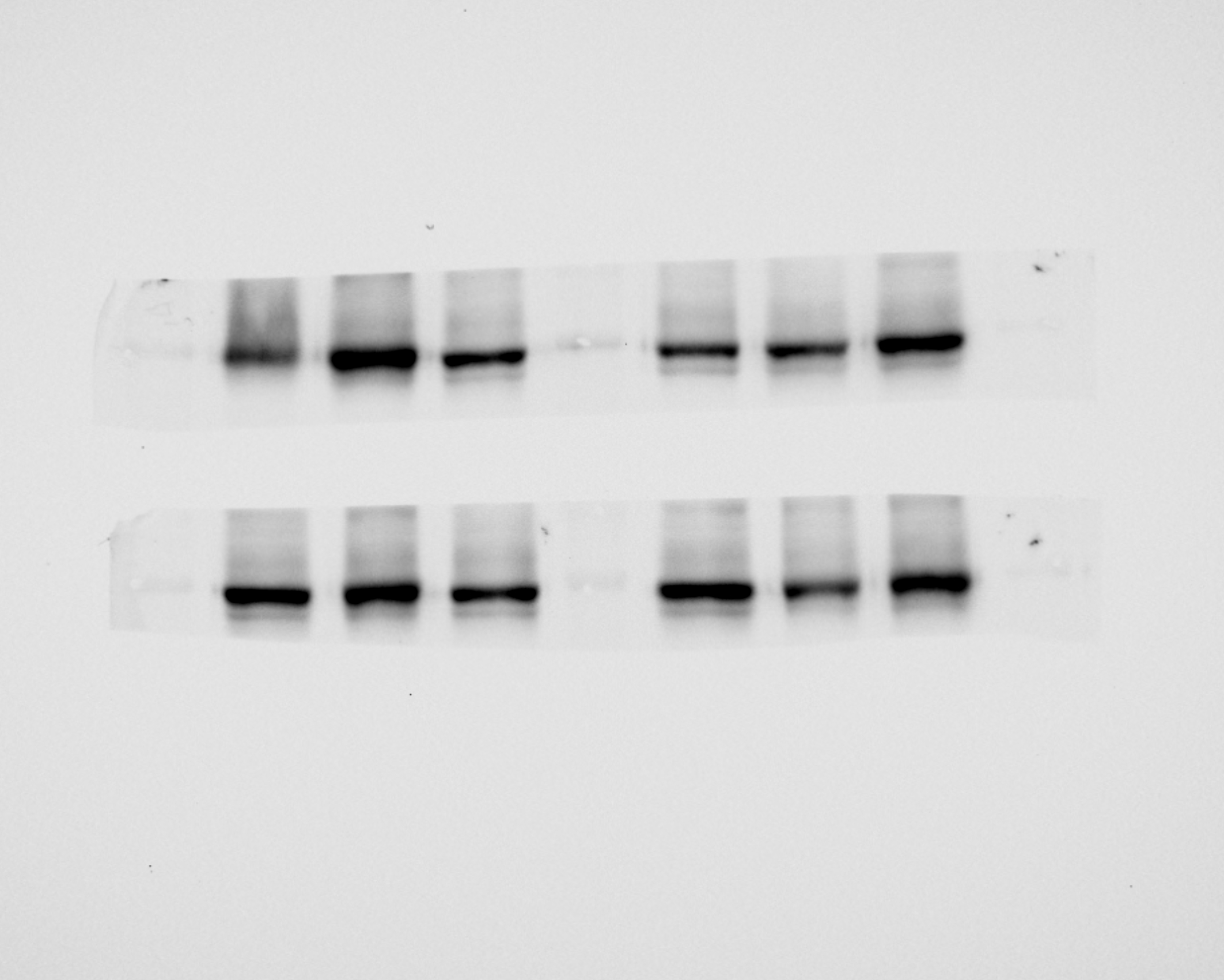

Supplement: Figure 5—figure supplement 1—source data 1. [file elife-90333-fig5-figsupp1-data1.zip › Figure 5-figure supplement 1 source data 1/Set 4/Set 4 p-AKT.jpg]
