# Supplementary material for: Smith–Magenis syndrome protein RAI1 regulates body weight homeostasis through hypothalamic BDNF-producing neurons and neurotrophin downstream signalling
Source: eLife. 2023 Nov 13;12:RP90333. doi: 10.7554/eLife.90333 (PMC10642964; doi:10.7554/eLife.90333)

Uncropped western blots for staining against antibodies for P-AKT & AKT. The membrane was stripped to stain for AKT after staining for p-AKT. Before staining, the membrane was cut at band 72KDa and below 53 KDa. The yellow box represents the cropped region for images shown in this manuscript.

Set 1 & 2: Figure 5—figure supplement 1

p-AKT

~60kD

~60kD

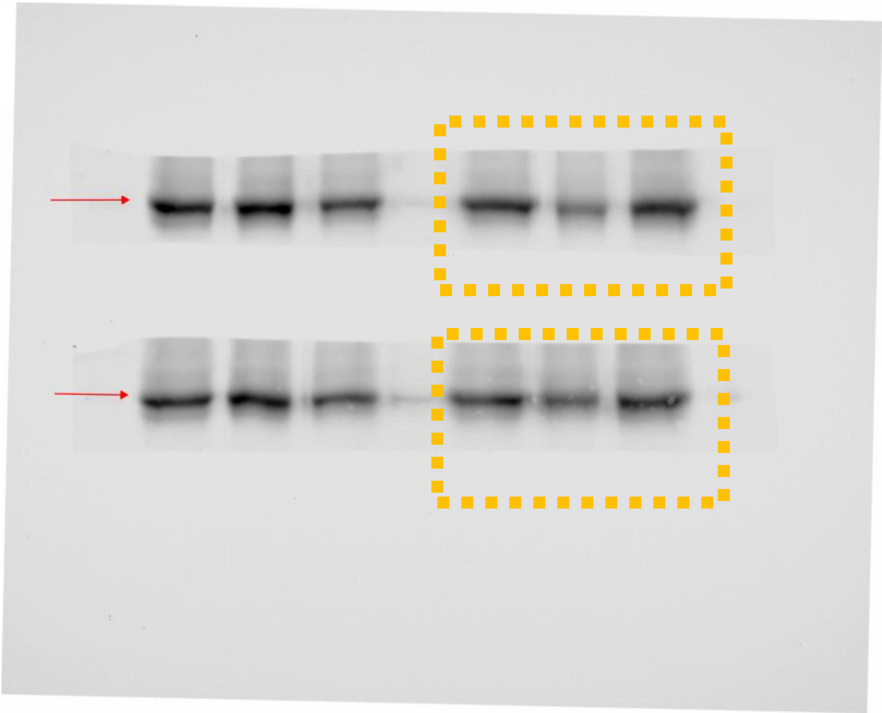

Set 1

Set 2

AKT

~60kD

~60kD

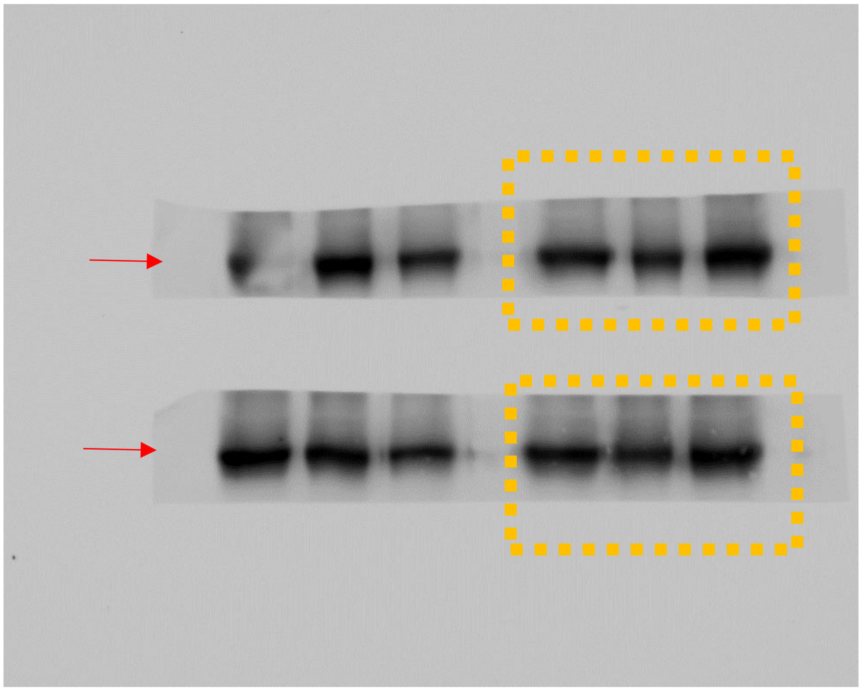

Set 1

Set 2

Figure 5A & Set 3: Figure 5—figure supplement 1

p-AKT

~60kD

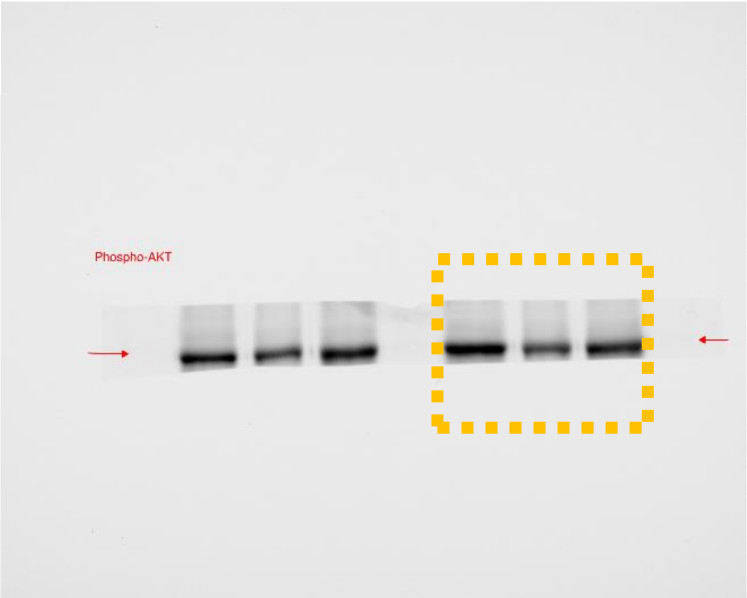

Set 3

AKT

~60kD

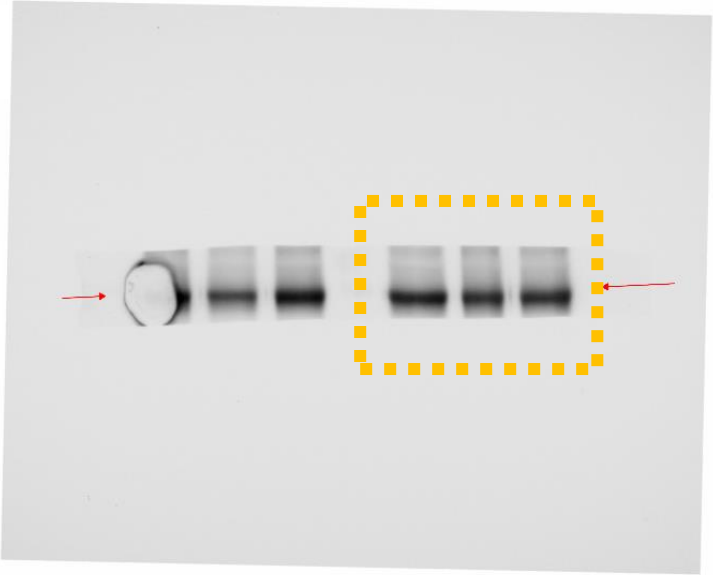

Set 3

Set 4: Figure 5—figure supplement 1

p-AKT

Set 4

~60kD

~60kD

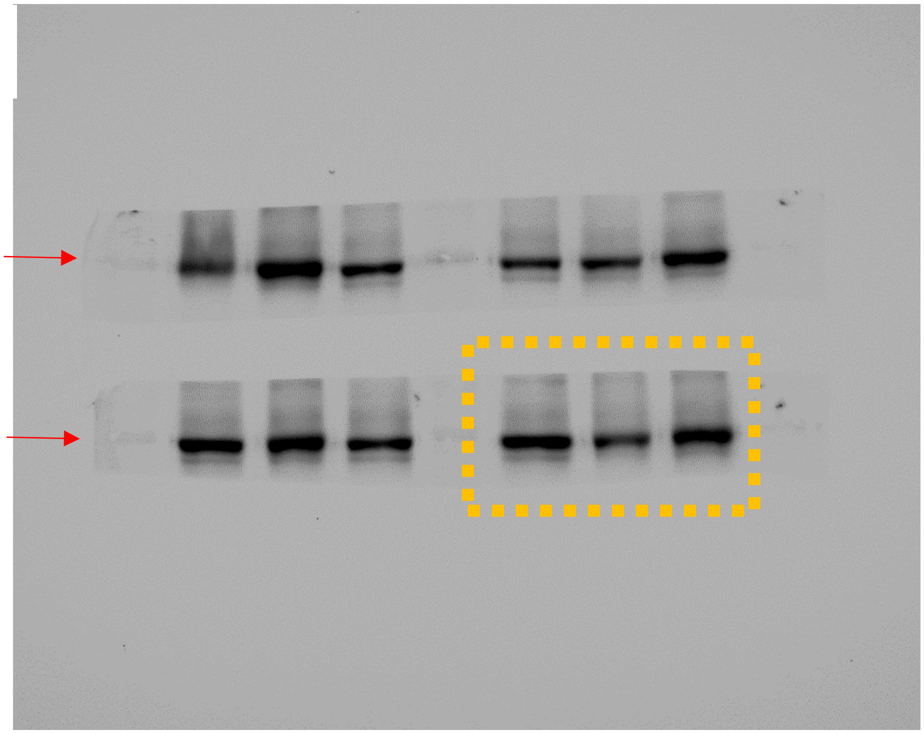

AKT

Set 4

~60kD

~60kD

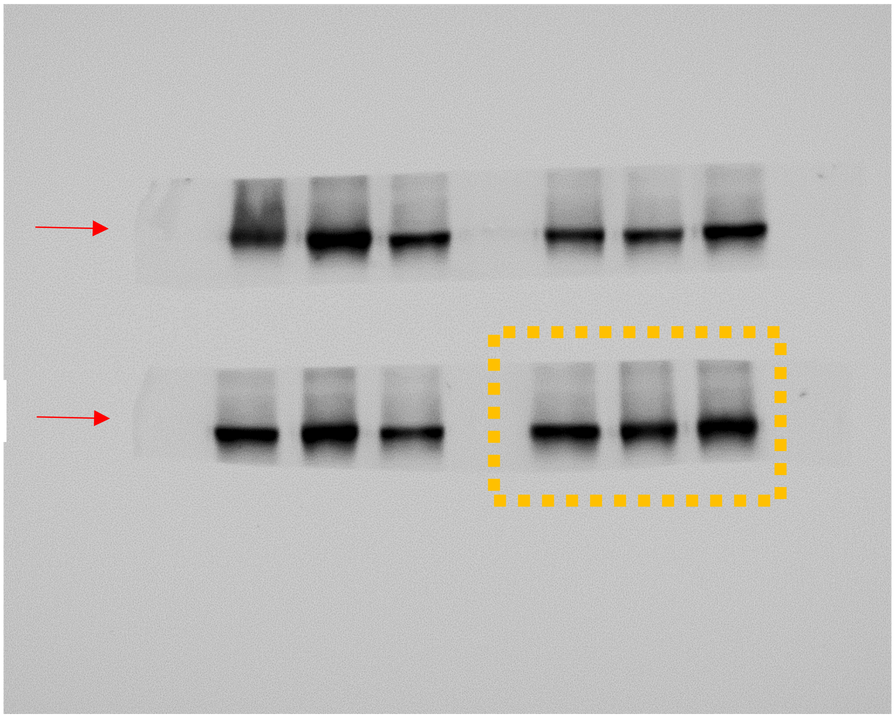

Supplement: Figure 5—figure supplement 1—source data 1. [file elife-90333-fig5-figsupp1-data1.zip › Figure 5-figure supplement 1 source data 1/Figure 5-figure supplement 1 source data 1.pdf]
